# Genetic Ablation of C/EBPα-p300 Pathway Blocks Development of Obese Pregnancy Associated Liver Disorders in Offspring

**DOI:** 10.1016/j.jcmgh.2023.11.006

**Published:** 2023-11-14

**Authors:** Margaret A. Hanlon, Ruhi Gulati, Michael Johnston, Yasmeen Fleifil, Maria Rivas, Nikolai A. Timchenko

**Affiliations:** 1 Division of General and Thoracic Surgery, Cincinnati Children’s Hospital Medical Center, Cincinnati, Ohio; 2 Department of Surgery, University of Cincinnati, Cincinnati, Ohio; 3 Institute of Biosciences, University of São Paulo (USP) Cidade Universitária, Butantã, São Paulo, Brazil

**Keywords:** Maternal Obesity, Fibrosis, Steatosis, Proliferation, High Fat Diet, C/EBPα

## Abstract

**Background & Aims:**

The obesity-associated nonalcoholic fatty liver disease represents a common cause of pediatric liver diseases, including the pediatric liver cancer hepatoblastoma. The mechanisms behind the development of fatty liver in children are not yet known. We examined the role of the C/EBPα-p300 pathway in the development of maternal obesity-associated fatty liver phenotype in offspring.

**Methods:**

Because the ability of C/EBPα to promote fatty liver phenotype is enhanced by CDK4-mediated phosphorylation of C/EBPα at Ser193 and subsequent formation of C/EBPα-p300 complexes, we used wild-type (WT) and C/EBPα-S193D and C/EBPα-S193A mutant mice to study the effects of maternal high-fat diet (HFD) on the liver health of offspring. The females of these mouse lines were fed an HFD before mating, and the pups were further subjected to either an HFD or a normal diet for 12 weeks.

**Results:**

WT female mice on the HFD before and during pregnancy and their subsequent offspring on the HFD had severe fatty liver, fibrosis, and an increased rate of liver proliferation. However, the HFD in C/EBPα-S193A mice did not cause development of these disorders. In HFD-HFD treated WT mice, C/EBPα is phosphorylated at Ser193 and forms complexes with p300, which activate expression of genes involved in development of fatty liver, fibrosis, and proliferation. However, S193A-C/EBPα mice do not have complexes of C/EBPα-S193A with p300, leading to a lack of activation of genes of fatty liver, fibrosis, and proliferation. The mutant C/EBPα-S193D mice have accelerated cdk4-dependent pathway and have developed steatosis at early stages.

**Conclusions:**

These studies identified the epigenetic cause of obese pregnancy–associated liver diseases and suggest a potential therapy based on inhibition of cdk4-ph-S193-C/EBPα-p300 pathway.

## Graphical abstract

**Figure:**
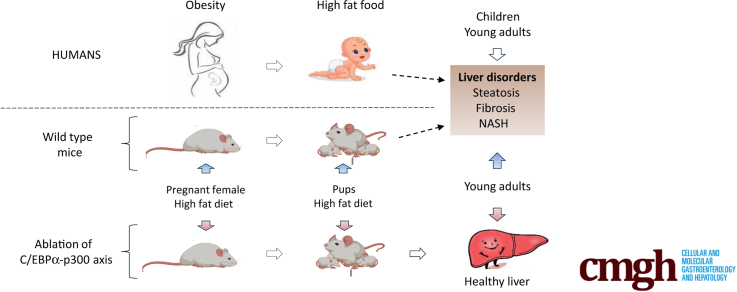

**Summary**

Obese and overweight expectant mothers lead to the development of fatty liver in their children. We found that activation of C/EBPα-p300 pathway is the cause of fatty liver in offspring and that ablation of this pathway prevents fatty liver.

Nonalcoholic fatty liver disease (NAFLD) is a dangerous liver disease that affects many people worldwide and has recently become the second highest cause of liver resections.^1^ Although investigations of NAFLD in adults have significantly improved our knowledge of underlying mechanisms, little is known about the pathways causing a fatty liver phenotype and fatty liver–mediated disorders in children. For a long time, the limitation of fatty liver studies in pediatric patients was related to the fact that fatty liver disorders are underdiagnosed in many young people because of mild or no clinical symptoms at the early stages. As a result, many cases of early stage NAFLD in children have not been reported or investigated. However, several recent studies of large cohorts of young NAFLD patients showed a strong correlation between pregnant patients being overweight or obese (OWOB) and the subsequent development of liver disorders in their children. A cohort study of 97 cases in Mexico City showed that 17% of participants had NAFLD, suggesting a strong association between maternal OWOB before and during pregnancy and the development of NAFLD in adult offspring.^2^ A study of 182 OWOB children revealed that 73% of these patients had liver fibrosis, also associated with parental obesity.^3^ A recent report examined 1105 women and their children (median age, 8.2 years) and found a high risk of liver injury associated with perfluoroalkyl and polyfluoroalkyl substances mediated obesity.^4^ A review of NAFLD in children in Mexico has reported data showing the increase of metabolic-associated fatty liver diseases in children.^5^ Examination of obese children showed that about 70% have liver fibrosis at the time of diagnosis for NAFLD.^6^ Taken together, these and other recent reports^7^, ^8^, ^9^, ^10^ reveal a strong correlation between overweight expectant mothers and the subsequent development of NAFLD and other liver disorders, including fibrosis and hepatocellular carcinoma in their children. These studies exhibit an urgent need for the understanding of molecular mechanisms by which obesity or a high-fat diet (HFD) causes NAFLD and other liver disorders in adolescents and young adults.

In this regard, several studies in animal models with a maternal fat intake demonstrate that the development of a fatty liver phenotype is the cause of severe liver diseases in offspring.^11^, ^12^, ^13^, ^14^ Our lab has been investigating mechanisms of NAFLD in adults and in aged animal models for over 20 years, and we have identified critical molecular pathways that cause NAFLD in the livers of adult patients and animals. One of these pathways is associated with C/EBPα, a master regulator protein in liver biology. C/EBPα is a transcription factor involved in a variety of activities including supports liver function, promotes liver quiescence, and protects the liver from development of cancer.^15^ This broad range of C/EBPα functions is associated with different post-translational modifications of C/EBPα in different biological settings. We found that Ser193 (Ser190 in human C/EBPα) is a key amino acid that controls the activities of C/EBPα.^16^, ^17^, ^18^ Particularly, it has been shown that ph-Ser193-C/EBPα is an essential player in the development of NALFD in adult livers.^16^^,^^19^^,^^20^ We also reported that a cyclin dependent kinase CDK4 is an enzyme that phosphorylates C/EBPα at Ser193, and that activation of CDK4 by cyclin D1/3 is involved in the development of NAFLD in adults.^16^^,^^19^^,^^20^

In relation to C/EBPα’s role in utero and after birth, a recent study demonstrated that C/EBPα is implicated in the caffeine-dependent development of a fatty liver phenotype in the offspring of obese mothers.^21^ Because of this report and our previous data targeting adults,^19^^,^^20^ we investigated the involvement of the ph-S193-C/EBPα-p300 pathway in the development of HFD pregnancy-associated liver disorders in offspring using a genetically modified mouse model, C/EBPα-S193A. We found that the mutation of Ser193 to Ala changes the biological activities of C/EBPα by altering protein-protein interactions, blocking the development of fatty liver, fibrosis, and proliferation in offspring. The underlying mechanisms are associated with the C/EBPα-S193A mutation preventing the interaction of C/EBPα with p300, blocking the activation of genes involved in the development of fatty liver, fibrosis, and increased liver proliferation.

## Results

### Fatty Liver Phenotype and Fibrosis Are Observed in Patients With Pediatric Liver Cancer

**Figure 1:**
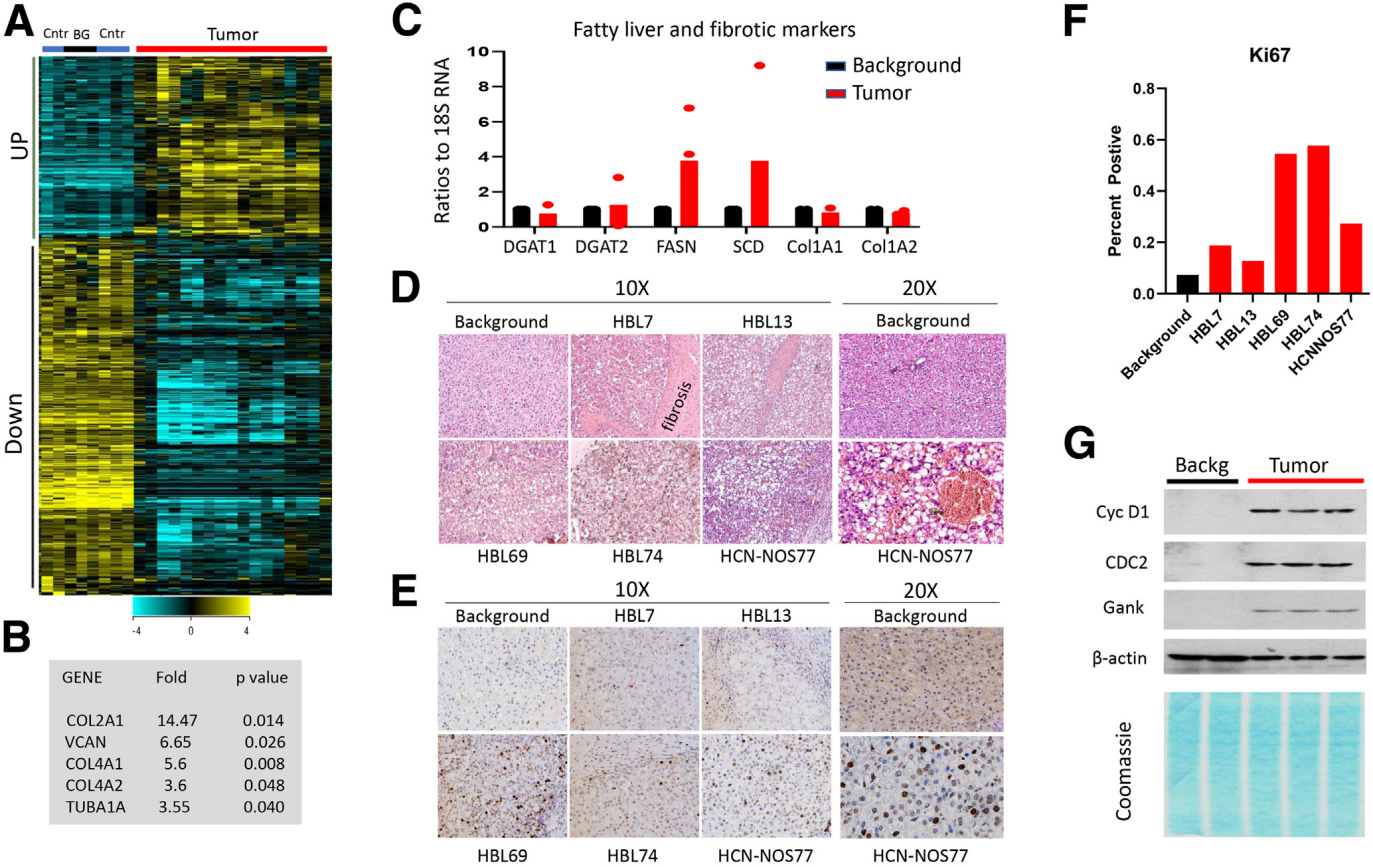
**Patients with pediatric liver cancer have fatty liver phenotype, fibrosis, and increased liver proliferation.** (*A*) Heat map of RNA sequencing results with archived Biobank of HBL samples. (*B*) List of fibrotic genes that are elevated in HBL. (*C*) Quantitative real-time polymerase chain reaction results of gene expression in a fresh Biobank of HBL patients (n = 3). (*D*) Examples of fatty liver phenotype in samples of a fresh Biobank. H&E staining shows accumulation of fat droplets under ×10 magnifications. *Right images* show pictures taken under ×20 magnifications. (*E*) ki67 staining of HBL with fatty livers (original magnification, ×10 and ×20). (*F*) Percentage of ki67-positive hepatocytes. (*G*) Expression of cell cycle proteins in livers of patients with fatty liver phenotype. Picture is a representative result of 2 replicates. Coomassie staining shows loading and integrity of the proteins.

Initial stages of fatty liver disorders in children go undiagnosed because of a lack of symptoms. This lack of symptoms, as well as a lack of specimens from young children with NAFLD, raised the question whether these early stages exist in children. Although the correlation of prenatal obesity with liver disorders in young children has been described,^2^^,^^3^ there are no direct experimental studies showing that NAFLD is observed in livers of young children. To shed light on this issue, we performed molecular analysis of available specimens from patients with hepatoblastoma (HBL). Our previous global RNA sequencing analyses of an archived HBL Biobank revealed dramatic changes in the gene expression of these samples.^22^^,^^23^ However, this global analysis did not display rare fatty liver cases representing patients who had a fatty liver phenotype (Figure 1*A*). Therefore, a closer investigation of 64 archived HBL cases was performed, identifying several HBL cases with fatty liver characteristics, including the elevation of fatty liver markers, fibrotic markers, and liver proliferation markers. Figure 1*B* demonstrates the fibrotic genes elevated in many cases of the archived Biobank. To further investigate the fatty liver phenotype in HBL patients, 21 samples in the fresh Biobank were collected and examined for fatty liver pathways. Quantitative real-time polymerase chain reaction analysis revealed that 5 HBL samples had elevated fatty liver and fibrotic markers (Figure 1*C*). H&E and ki67 staining revealed elevated fat droplets and a dramatic increase in liver proliferation in the tumor sections of these HBL patients, relative to background (adjacent) regions (Figure 1*D–F*). Western blotting with cell cycle proteins showed a dramatic elevation of cyclin D1, cdc2, and cancer-associated protein Gankyrin (Figure 1*G*). Thus, these molecular studies of available specimens identified a group of HBL patients (children) who have characteristics of fatty liver diseases at age 1–3 years. These data provided a rationale for testing the hypothesis that the development of fatty liver phenotype in young children might be a critical event in the subsequent development of liver disorders in young adults.

### Treatment of Pregnant WT Mice With HFD Causes a Severe Fatty Liver Phenotype in Offspring, Whereas the Treatment of Pregnant S193A-C/EBPα Mice With HFD Does Not

**Figure 2:**
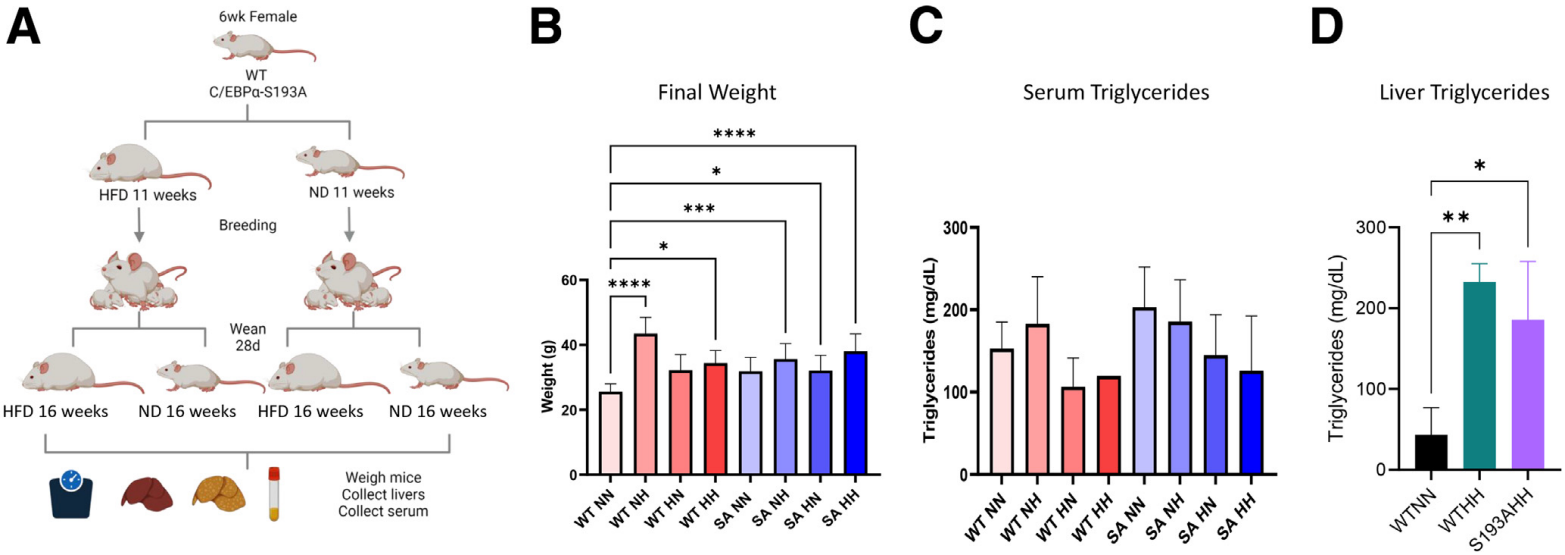
**Studies of maternal obesity associated liver diseases in offspring.** (*A*) Strategy for investigations of consequences of obesity-pregnant females in offspring. Detailed description of the strategy can be found in “Methods”. (*B*) Examination of final weight of mice in 4 diet arms of WT and C/EBPα-S193A mice (n = 2–8 animals per arm). One-way analysis of variance was used to do multiple comparisons. WT NN vs WT NH (*P* < .0001), WT NN vs WT HH (*P* = .0273), WT NN vs S193A NH (*P* < .0001), WT NN vs S193A HN (*P* = .0295), and WT NN vs S193A HH (*P* < .0001) were statistically significant. (*C*) Levels of TGs in serum (n = 3–12 animals per arm). One-way analysis of variance was used to do multiple comparisons; however, there was no significance between any of the arms. (*D*) Levels of TGs in livers (n = 3 animals per arm). One-way analysis of variance was used to do multiple comparisons. WT NN vs WT HH (*P* = .0051) and WT NN vs S193A HH (*P* = .0191) were both statistically significant.

Because the critical role of ph-S193-C/EBPα-p300 pathway in HFD-mediated fatty liver of adult mice,^19^^,^^20^ we asked whether this pathway is involved in maternal obesity-mediated liver disorders observed in offspring. For this goal, we used wild-type (WT) mice and C/EBPα-S193A mice. The S193A (Ser to Ala) mutation reduces interactions of C/EBPα with p300.^19^^,^^20^^,^^24^ Figure 2*A* demonstrates the strategy used for feeding pregnant females with HFD and subsequent investigations of liver disorders in offspring. Female breeders of WT and C/EBPα-S193A mice were fed with HFD for 6 weeks before breeding. At weaning (28 days), pups were separated into 2 arms; one arm was treated with a normal diet, and the second arm was treated with HFD. Animals were killed at 16 weeks, and liver tissues were investigated with the focus on fatty liver phenotype, fibrosis, and proliferation. Thus, 4 arms of study were generated for each mouse line: normal to normal diet (NN), normal to high-fat diet (NH), high-fat to normal diet (HN), and high-fat to high-fat diet (HH). One-way analysis of variance of final body weight found significant differences between WT NN vs WT NH, WT HH, SA NH, SA HN, and SA HH (Figure 2*B*). Statistical analysis of serum triglyceride (TG) levels did not determine any significant differences (Figure 2*C*). However, we observed statistically significant elevation of liver TGs in the WT HH arm and the C/EBPα-S193A HH arm compared with levels of TGs in WT NN arm. Statistical analyses indicated that there are no differences between the WT HH arm vs C/EBPα-S193A HH arm.

**Figure 3:**
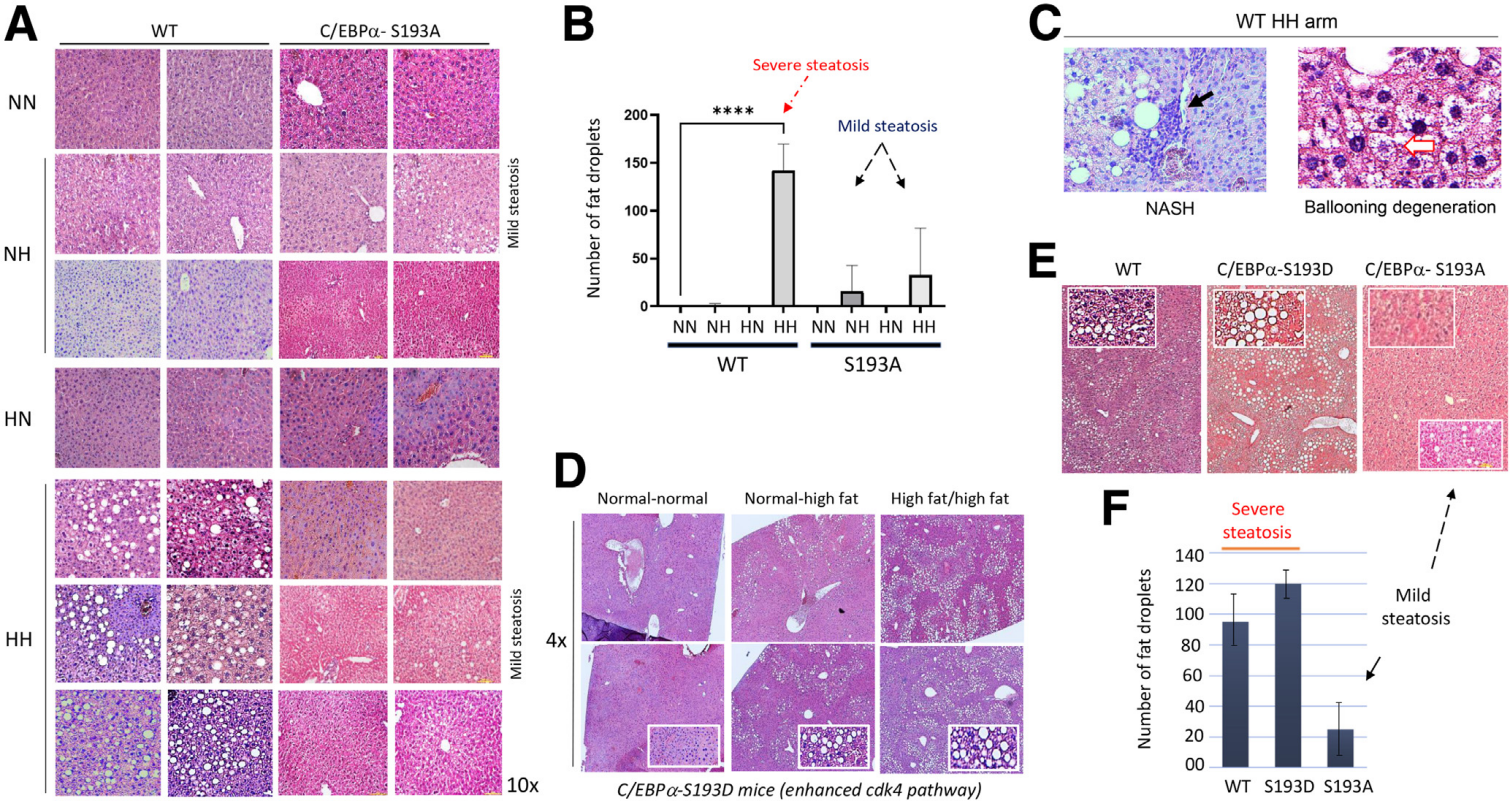
**Genetic ablation of C/EBPα-p300 pathway blocks development of steatosis in offspring of HFD-treated pregnant females.** (*A*) H&E staining of 4 diet arms of WT and C/EBPα-S193A mice. Representative images for 2 mice of NN and HN arms and for 4–6 mice of NH and HH arms are shown under ×10 magnification. (*B*) Calculations of number of macrovesicular fat droplets per field (×10 magnification) in livers of 4 diet arms of WT and C/EBPα-S193A mice. *Bar graphs* show results of calculations in 10 fields from livers of 3 mice of each genotype. Only small size fat droplets (mild steatosis) were found in some regions of livers of C/EBPα-S193A mice. (*C*) Images of inflammation and hepatocytes with ballooning degeneration observed in livers of HH arm of WT mice. (*D*) H&E staining of 3 diet arms of C/EBPα-S193D mice: NN diet arm, NH fat diet arm, and HH diet arm. Pictures under ×4 magnification are shown. ×20 magnifications are shown in *white squares*. (*E*) Typical images of livers of HH arms of WT mice, C/EBPα-S193D, and C/EBPα-S193A mice under ×4 magnification. ×20 magnifications are shown in *white squares*. Fat droplets in C/EBPα-S193D mice have a bigger size. (*F*) *Bar graphs* show average number of fat droplets per field. No statistically significant differences were found for WT and C/EBPα-S193D mice. Counting of small size fat droplets of C/EBPα-S193A mice is also shown.

Although blood parameters showed minor differences between WT and C/EBPα-S193A mice, a dramatic difference in the fatty liver phenotype was observed in the HH arm of WT and C/EBPα-S193A mice. H&E staining revealed that the livers of WT and C/EBPα-S193A mice have small and identical amounts of fat droplets in offspring in the NH and HN diet arms. However, the HH arm shows the development of a severe fatty liver phenotype in WT mice (Figure 3*A*). In comparison, examination of 12 HH C/EBPα-S193A mice showed no steatosis in 8 animals and mild steatosis in 3 animals, whereas 1 mouse showed steatosis compared with the WT HH arm (Figure 3*A*). Interestingly, a similar range of inhibition of steatosis was found in the NH arm of C/EBPα-S193A mice. Examination of 7 C/EBPα-S193A mice of the NH arm found no steatosis in 4 animals and a minor accumulation of fat droplets in some areas of the livers of 3 mice (Figure 3*A*). To get a better understanding of the development and inhibition of steatosis, we counted the number of fat droplets per field (×10 magnification) in the 4 diet arms of WT and C/EBPα-S193A mice. Figure 3*B* shows results of these studies. No fat droplets were found in the NN and HN arms of both WT and C/EBPα-S193A mice. No or few fat droplets per field (2–8) were found in the NH arms of both WT and C/EBPα-S193A mice. One hundred twenty to 150 macrovesicular droplets (severe steatosis) were found in livers of the HH arm of WT mice; however, no macrovesicular droplets were observed in livers of HH arm of C/EBPα-S193A mice. Instead, we found that livers of 3 C/EBPα-S193A mice in the HH arm had 2–3 areas containing small fat droplets, which we defined as mild steatosis. To study the development of this mild steatosis, we counted the small fat droplets in the livers of C/EBPα-S193A mice. In Figure 3*B*, up to 50 small fat droplets were found in some areas of the livers, whereas other fields had no fat droplets. Taking these studies together, we conclude that severe steatosis is developed in the HH arm of WT mice, but development of severe hepatic steatosis is inhibited in the HH arm of C/EBPα-S193A. Regarding the mild steatosis in livers of C/EBPα-S193A mice, further molecular analyses did not find differences in pathways of fatty liver in these mice. This is related to the fact that mild steatosis was observed in small local areas, whereas molecular analyses were performed with whole livers. In addition, we have found that WT mice of the HH arm have characteristics of nonalcoholic steatohepatitis (NASH), such as inflammation and hepatocytes with ballooning degeneration (Figure 3*C*). These abnormalities were not observed in the HH arm of all C/EBPα-S193A mice.

The NH diet arms of WT mice and C/EBPα-S193A mice did not develop steatosis after treatments. Because our previous studies showed that adult mice with the S193D mutation (which mimics cdk4-dependent phosphorylation of S193) are more sensitive to HFD,^19^^,^^25^ we performed treatments of these mice using our protocol of prenatal HFD and focused on the NN, NH, and HH arms. H&E staining of the livers of CEBPα-S193D mice revealed that they develop steatosis in the NH arm and severe steatosis in the HH arm (Figure 3*D*). Figure 3*E* presents the typical images of fat droplets in WT, S193D, and S193A mice taken under ×4 magnifications, which provide information about steatosis in large areas of the livers. Analyses of these areas revealed that livers in the HH arm of C/EBPα-S193D contained a higher number of fat droplets per field, and that the size of the droplets was bigger than in the livers of WT mice (Figure 3*E*, enlarged squares). To get a better understanding of these differences, we counted the number of fat droplets. A bar graph of Figure 3*F* shows that there are no significant differences in the number of fat droplets between WT and C/EBPα-S193D mice; however, the size of fat droplets is larger in livers of C/EBPα-S193D mice. Taking together the studies in WT mice, C/EBPα-S193A, and C/EBPα-S193D mice, we conclude that the phosphorylation of C/EBPα at Ser193 is a critical event in the development of obese pregnancy–mediated liver steatosis and NASH in offspring.

### Inhibition of Fatty Liver Phenotype in the HH Arm of C/EBPα-S193A Mice Is Associated With Lack of C/EBPα-P300 Complexes and Subsequent Low Levels of Enzymes of TG Synthesis

**Figure 4:**
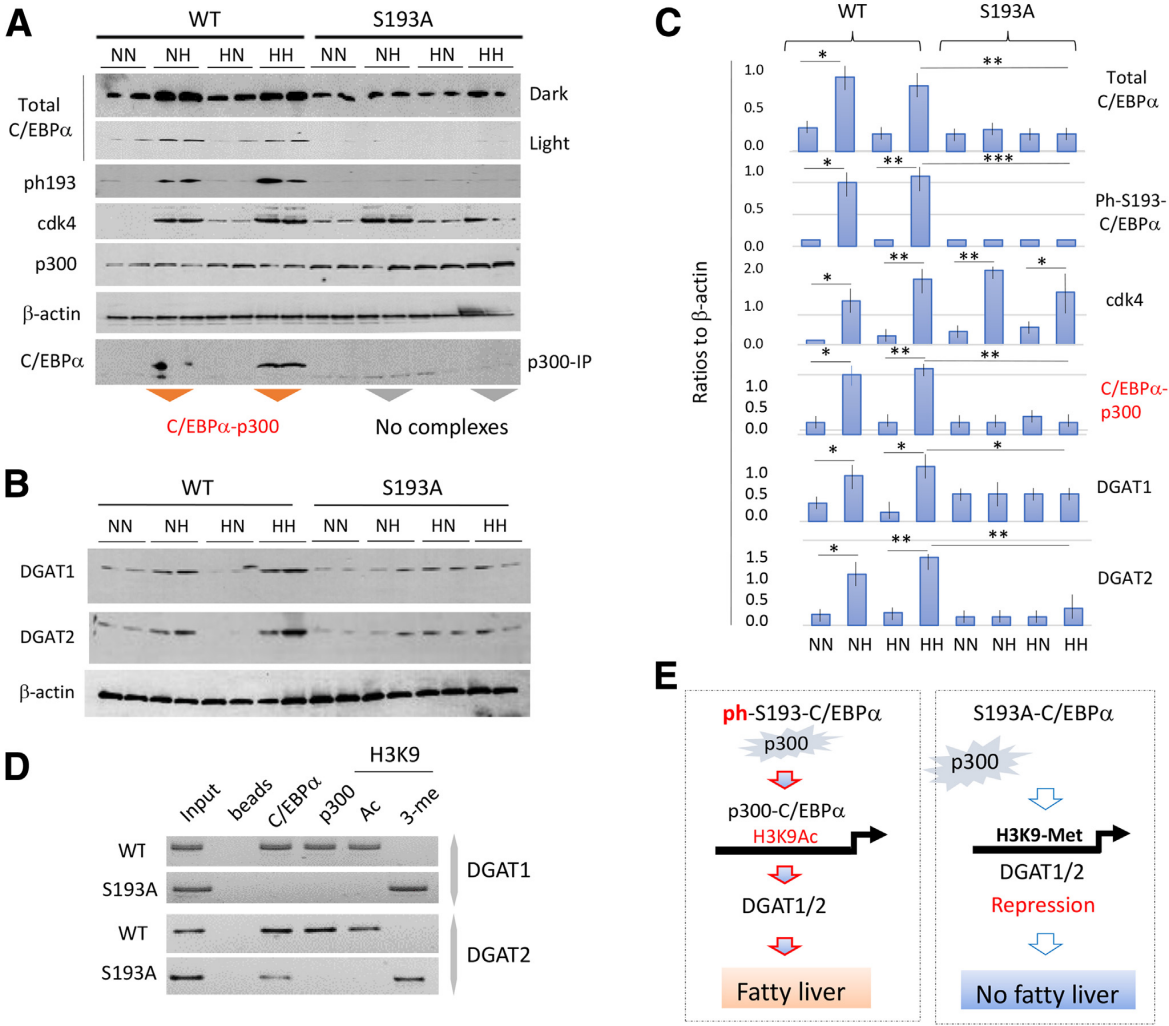
**Inhibition of fatty liver pathways in livers of C/EBPα-S1983A mice.** (*A*) C/EBPα-p300 pathway is eliminated in C/EBPα-S193A mice. *Upper panels* show Western blotting with antibodies to ph-S193-C/EBPα, total C/EBPα, cdk4, and p300. *Bottom image* shows p300 IP and subsequent Western blot with antibodies to C/EBPα. Two animals of each arm were examined. (*B*) Expression of DGAT1 and DGAT2 was examined by Western blotting. Two animals of each arm were examined. (*C*) Levels of proteins and levels of C/EBPα-p300 complexes shown on *A* and *B* were calculated as ratios to β-actin. ∗∗PI < .0001; ∗PI < .001. ImageJ software was used for these calculations. (*D*) ChIP assay of DGAT1 and DGAT2 promoters using chromatin solutions from HH arms of WT and C/EBPα-S193A mice. Ac, acetylated H3K9; B, beads; In, input; Me, trimethylated H3K9. (*E*) Summary of studies showing molecular basis for inhibition of obese pregnancy–associated steatosis in offspring of C/EBPα-S193A mice.

Because WT and C/EBPα-S193D mice have identical development of steatosis in HH arms, we focused our molecular studies to compare WT and C/EBPα-S193A mice. To understand the underlying molecular mechanisms of NASH prevention in C/EBPα-S193A mice, a set of molecular biology studies was conducted. Because C/EBPα-S193A mice have only one single amino acid substitution in the genome, our studies were focused on the pathways downstream of WT C/EBPα that are affected by this substitution. Our previous studies showed that WT C/EBPα interacts with histone acetyltransferase p300 and increases levels of TG, the main components of fat droplets, by binding to promoters of enzymes of TG synthesis.^19^^,^^20^^,^^25^ Therefore, we examined this pathway in the 4 arms of WT and C/EBPα-S193A mice. Western blotting showed an elevation of total C/EBPα in the NH and HH arms in WT mice, whereas levels of C/EBPα in C/EBPα-S193A mice were not altered (Figure 4*A* and *C*). Note that we have previously found that total levels of C/EBPα are lower in C/EBPα-S193A mice because of the disruption of an auto-regulation loop.^17^^,^^18^ Consistent with this finding, levels of C/EBPα-S193A protein in the mutant mice were also lower than in WT mice in all 4 arms (Figure 4*A* and *C*). We next investigated expression of CDK4, the enzyme that phosphorylates C/EBPα at Ser193,^16^^,^^19^^,^^20^ and found that CDK4 is elevated in the NH and HH arms of WT mice, as well as in the NH and HH arms of C/EBPα-S193A mice. Quantitation and statistical analyses showed minor differences in the levels of elevation of cdk4 between diet arms, but in all cases levels of cdk4 were high (Figure 4*C*). The elevation of CDK4 in livers of S193A-C/EBPα mice showed that the subsequent block of pathologic changes is associated with the S193A mutation. Examination of phosphorylation of C/EBPα at Ser193 by Western blot with specific antibodies showed a dramatic elevation of ph-S193-C/EBPα in the WT HH arm and a weaker, but detectable, elevation of ph-S193-C/EBPα in NH arm of WT mice (Figure 4*A* and *C*). Although CDK4 is elevated in the NH and HH arms of C/EBPα-S193A mice, the ph-S193 isoform is not detected in S193A mice because of the mutation of Ser193 to Ala. Co-immunoprecipitation studies revealed that ph-S193-C/EBPα forms complexes with p300 in NH and HH arms of WT mice (Figure 4*A*, p300 immunoprecipitation and Figure 4*C*). Expression of DGAT1 and DGAT2, direct targets of the C/EBPα-p300 complexes,^16^, ^17^, ^18^, ^19^, ^20^^,^^24^^,^^25^ were examined by Western blot, finding an increase of these enzymes in the NH and HH arms in WT mice that also had high levels of C/EBPα-p300 complexes (Figure 4*B* and *C*). Chromatin immunoprecipitation (ChIP) studies demonstrated that the C/EBPα-p300 complexes occupy DGAT1 and DGAT2 promoters in HH arm of WT mice, whereas p300 is not detected on these promoters in HH arm of C/EBPα-S193A mice (Figure 4*D*). Figure 4*E* summarizes molecular studies of fatty liver in WT and S193A mice. Taken together, we found that the lack of fatty liver phenotype in the HH arm of C/EBPα-S193A mice is mediated by the genetic mutation Ser193 to Ala and subsequent lack of C/EBPα-p300 complexes, leading to a failure to increase levels of enzymes of TG synthesis.

### Genetic Ablation of C/EBPα-P300 Pathway Blocks Development of Fibrosis in Offspring of High-Fat Diet/High-Fat Diet Treated C/EBPα-S193A Mice

**Figure 5:**
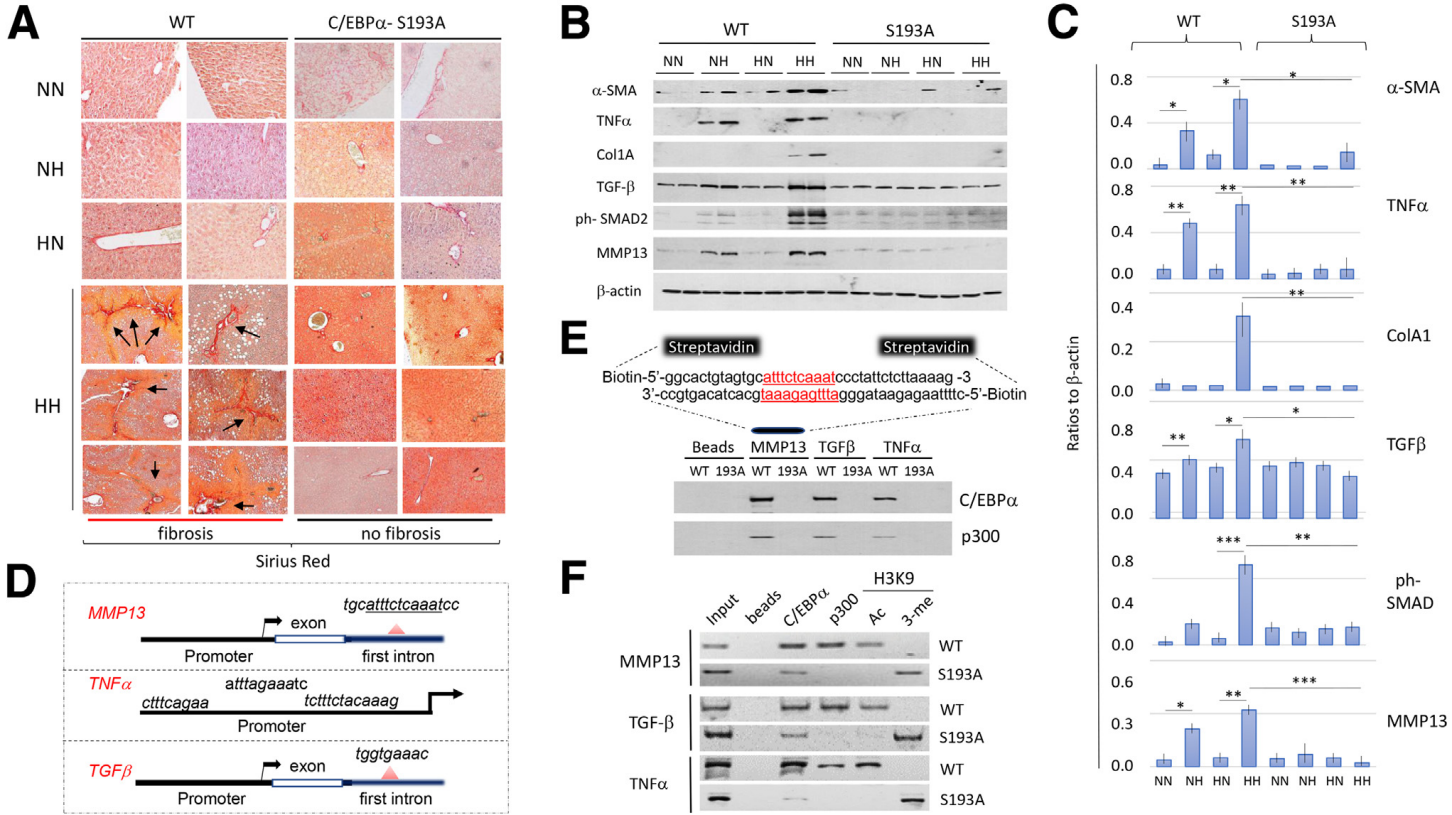
**Obese pregnancy–mediated fibrosis is inhibited in offspring of C/EBPα-S193A mice.** (*A*) Sirius Red staining of 4 arms of WT and C/EBPα-S193A mice. (*B*) Expression of fibrotic genes α*-SMA*, *TNF*α, *Col1A1*, *MMP13*, *TGFβ,* and its target ph-Ser465/467-SMAD2 in 4 diet arms of WT and C/EBPα-S193A mice. Two animals of each arm were examined. (*C*) Levels of fibrotic proteins shown in *B* were calculated as ratios to β-actin. ∗∗PI < .0001; ∗PI < .001. (*D*) Identification of C/EBPα consensuses in *MMP13*, *TNF*α, and *TGFβ* genes. (*E*) Pull-down assay showing interactions of C/EBPα-p300 complexes with promoters/introns of *MMP13*, *TNF*α, and *TGFβ* genes. Representative picture of 2 replicates is shown. (*F*) Representative result of ChIP assay with promoters/first introns of fibrotic genes. Chromatin solutions of HH arms of WT mice and C/EBPα-S193A mice were used. Ac, acetylated H3K9; B, beads; In, input; Me, trimethylated H3K9.

Because around 70% of obese children develop liver fibrosis at the time of diagnosis for NAFLD,^6^ we examined whether the offspring in the HH arm of WT mice develop fibrosis, and whether the elimination of the ph-S193-C/EBPα-p300 pathway in C/EBPα-S193A mice inhibits development of fibrosis. For this goal, livers of WT and S193A mice of all 4 diet arms were stained with Sirius Red. In WT mice, NN, NH, and HN arms showed no increase in Sirius Red staining; however, strong staining was found in the HH arm of WT mice (Figure 5*A*). Sirius Red staining of all 4 diet arms of C/EBPα-S193A mice showed no signs of fibrosis (Figure 5*A*). This result suggests that like the inhibition of fatty liver, the mutation of Ser193 to Ala blocks the development of fibrosis, mediated by prenatal and postnatal HFD. To further analyze molecular changes in WT and C/EBPα-S193A mice associated with fibrosis, we examined expression of fibrotic genes. Western blotting showed that fibrosis markers, α-SMA, TNFα, Col1A, MMP13, TGFβ, and its target ph-Ser465/467-SMAD2, are increased in WT mice in NH and HH arms, whereas livers of C/EBPα-S193A mice revealed no change to these proteins in any diet arm (Figure 5*B*). We next calculated levels of the proteins as ratios to a loading control (β-actin) and found statistically significant elevation of all of them in NH and HH arms of WT mice, but we did not find an increase of these proteins in NH and HH arms of C/EBPα-S193A mice (Figure 4*C*). Because of a similar pattern of elevation of fatty liver associated proteins DGAT1 and DGAT2 (Figure 4) and the fibrotic proteins (Figure 5), we suggested that fibrotic proteins might be under C/EBPα-dependent epigenetic control in the NH and HH arms of WT mice. Therefore, we examined promoters and first exon/intron regions of *MMP13*, *TNF*α, and *TGFβ* genes and identified perfect consensuses for C/EBPα in the promoter of *TNF*α gene, as well as in the first introns of *MMP13* and *TGFβ* (Figure 5*D*). To examine whether C/EBPα-p300 complex binds to these consensuses, we performed 2 sets of experiments, pull-down assay with biotinylated double-stranded oligomers containing C/EBPα consensuses and ChIP assay using chromatin solutions from the HH arms of WT and C/EBPα-S193A mice. The pull-down assay unveiled that the C/EBPα-p300 complex binds to all 3 oligomers in nuclear extracts from HH arm of WT mice, but no binding was observed in protein extracts from the HH arm of C/EBPα-S193A mice (Figure 5*E*). The ChIP assay revealed that C/EBPα-p300 complexes are bound to these consensuses in HH WT mice, whereas only C/EBPα is detected on the promoters/introns of these genes in C/EBPα-S193A mice (Figure 5*F*). The lack of p300 on these DNA regions is consistent with no acetylation of H3K9 and with the increase of H3K9 methylation. This pattern of H3K9 modifications shows a repression of the genes. Taken together, these studies showed that HFD during pregnancy causes fibrosis in the offspring of WT mice via the ph-S193-C/EBPα-p300 pathway, and that this pathway is eliminated in C/EBPα-S193A mice, leading to the inhibition of fibrosis. It is likely that the inhibition of fibrosis in the HH arm of C/EBPα-S193A mice might be associated with the lack of steatosis in these livers (Figure 3). It is also possible that both C/EBPα-S193A mutation and lack of steatosis contributed to the inhibition of fibrosis in the HH arm of C/EBPα-S193A mice.

### Prenatal Obesity-Mediated Liver Proliferation in Offspring Is Inhibited in C/EBPα-S193A Mice

**Figure 6:**
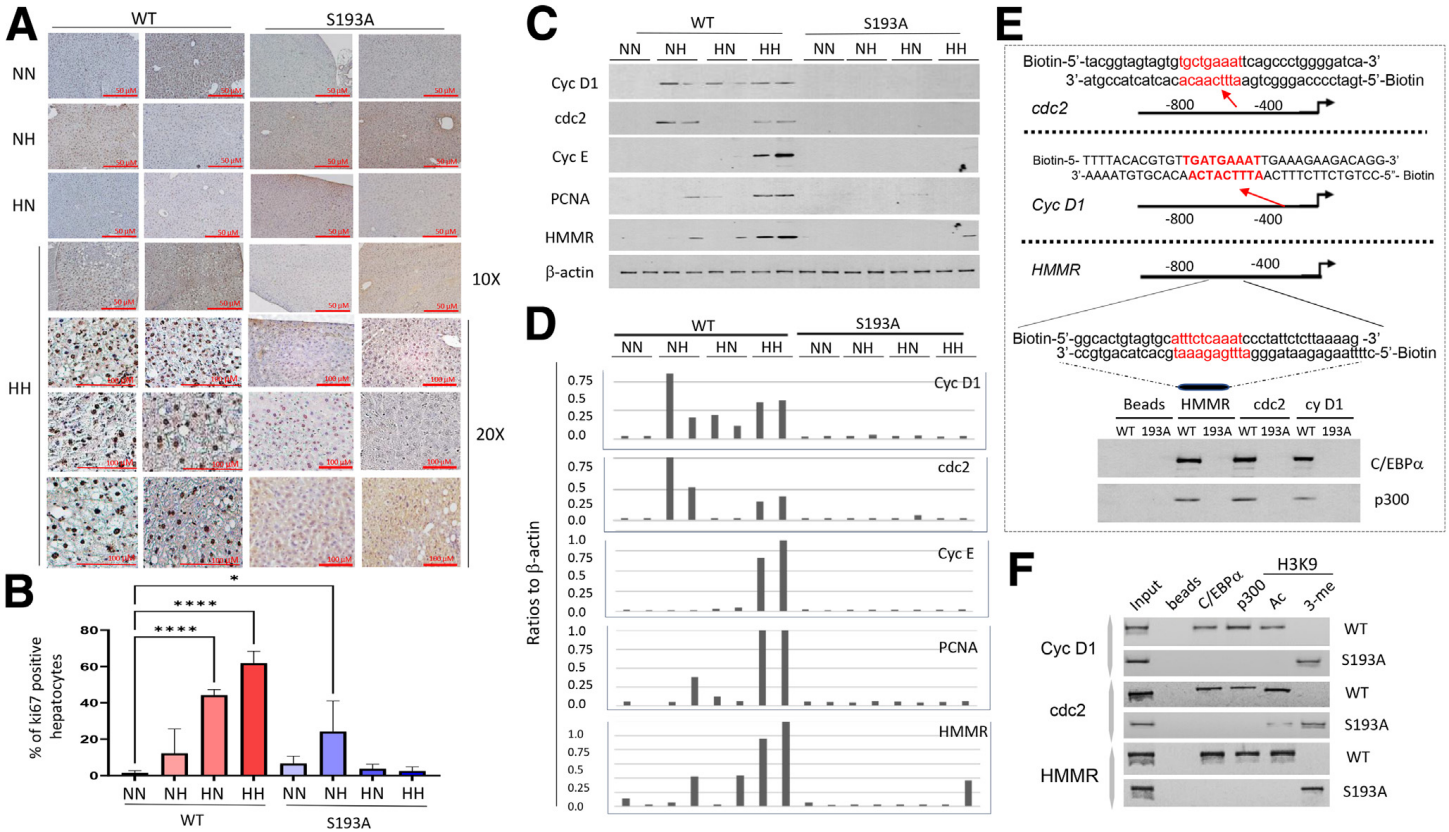
**Liver proliferation in offspring of obese-pregnant mice is inhibited in livers of C/EBPα-S193A mice.** (*A*) ki67 staining shows inhibition of liver proliferation in offspring of obese-pregnant mice. *Upper panel* shows images under ×10 magnification; *bottom panel* shows images under ×20 magnification. *Scale bars* are shown for each image. (*B*) *Bar graphs* show percentage of ki67-positive hepatocytes in each diet arm of WT and C/EBPα-S193A mice. One-way analysis of variance was used to do multiple comparisons. WT NN vs WT HN (*P* < .0001), WT NN vs WT HH (*P* < .0001), and WT NN vs S193A NH (*P* = .0158) were statistically significant. (*C*) Expression of cell cycle proteins was determined by Western blot. Two animals of each arm were examined. (*D*) Levels of cell cycle proteins were calculated as ratios to β-actin. (*E*) *Upper*: identification of C/EBPα consensuses in promoters of cell cycle genes. Structure/sequences of biotinylated double-stranded oligonucleotides are shown. *Bottom***:** pull-down assay shows interactions of C/EBPα-p300 complexes with promoters of HMMR, cdc2, and Cyc D1 genes. (*F*) ChIP assay shows that C/EBPα-p300 complexes occupy the promoters of HMMR, cdc2, and Cyc D1 genes in HH arm of WT mice but are absent on the promoters of these genes in HH arm of C/EBPα-S193A mice. Ac, acetylated H3K9; B, beads; Me, trimethylated histone H3K9.

In adult mice, C/EBPα is a strong inhibitor of proliferation under many biological settings including liver regeneration and fibrosis.^15^^,^^20^^,^^26^ However, an examination of liver proliferation by ki67 staining in our 4 diet arms found that livers of the HH arm in WT mice have a very high rate of proliferation in offspring (Figure 6*A*). It was also surprising that C/EBPα-S193A mice in the HH arm had almost complete inhibition of proliferation (Figure 6*A*). Calculations of ki67-positive hepatocytes showed that liver proliferation increased in WT mice in the NH, HN, and HH arms, with statistically significant levels in the HH arm. However, only the NH arm of C/EBPα-S193A mice showed a slight increase of proliferation, whereas other arms had no proliferation (Figure 6*B*). These observations suggested that under these conditions, WT C/EBPα is converted into the protein that promotes liver proliferation. In agreement with this suggestion, a recent article investigated activities of WT C/EBPα in utero and shortly after birth, finding that C/EBPα promotes liver proliferation and NAFLD in rats whose mothers were prenatally treated with caffeine.^23^ Moreover, another recent report presented evidence that C/EBPα promotes NASH and hepatocellular carcinoma via activation of hyaluronan mediated motility receptor (HMMR) by binding to its promoter.^27^

Because of our new observations and these reports demonstrating the proliferation promoting activities of WT C/EBPα, we have examined the hypothesis that a prenatal HFD changes the biological activity of C/EBPα and creates a new activity, increasing liver proliferation in offspring. Because of our results showing the lack of C/EBPα-p300 complexes in the livers of C/EBPα-S193A mice, the main theme of this hypothesis was that the new activity of C/EBPα might include activation of transcription of cell cycle genes via formation of complexes with p300. Therefore, we first examined the expression of cell cycle proteins and HMMR and found that in the HH arm of WT mice, cyclin D1, cdc2, cyclin E, PCNA, and HMMR were strongly elevated, whereas the NH arm of WT mice had low elevation of these proteins. On the contrary, these proteins are not detectable in any of the 4 diet arms of C/EBPα-S193A mice (Figure 6*C* and *D*). Given this result, we next searched the promoter regions of *HMMR*, *cdc2,* and *cyclin D1* genes and found perfect C/EBPα consensuses within these promoters. The C/EBPα consensuses are near the start of transcription of these genes (Figure 6*E*, upper). To examine whether C/EBPα-p300 complexes bind to these regions, we have synthesized biotinylated dsDNA oligomers with these C/EBPα consensuses and performed pull-down experiment using protein extracts from the HH arms of WT mice and C/EBPα-S193A mice as it is described for fibrotic genes. These studies showed a strong interaction of the C/EBPα-p300 complexes with the promoters in protein extracts from the HH arm of WT mice, but no interactions were found in protein extracts isolated from the HH arm of C/EBPα-S193A mice (Figure 6*E*, bottom). Consistent with these results, ChIP assay revealed that the promoters of *cyclin D1*, *cdc2*, and *HMMR* genes are occupied by the C/EBPα-p300 complexes in the WT HH arm, and that H3K9 is acetylated on these promoters, showing the active transcription from these genes (Figure 6*F*). In comparison, C/EBPα-p300 complexes are not found on these promoters in the HH arm of C/EBPα-S193A mice, and histone H3 is tri-methylated (Figure 6*F*). These studies revealed that a prenatal HFD causes liver proliferation in offspring via increase of C/EBPα-p300 complexes and subsequent activation of *cyclin D1*, *cdc2,* and *HMMR* via binding to their promoters. The increase of maternal HH-mediated proliferation in offspring is eliminated by genetic ablation of C/EBPα-p300 pathway. In addition, it is known that proliferation of the liver might be activated by liver injury.^26^ In this regard, it is likely that the severe fibrosis (liver injury) in the HH arm of WT mice promotes proliferation in the liver, and that the lack of fibrosis in the HH arm of C/EBPα-S193A mice contributes to the reduction of liver proliferation in offspring. Taken together, ph-S193-C/EBPα was found to be a key translator of HFD conditions linked with obesity during pregnancy that led to liver proliferation in offspring.

### Molecular Mechanisms of Inhibition of Obese Pregnancy–Associated Liver Diseases in Offspring of Mutant C/EBPα-S193A Mice

**Figure 7:**
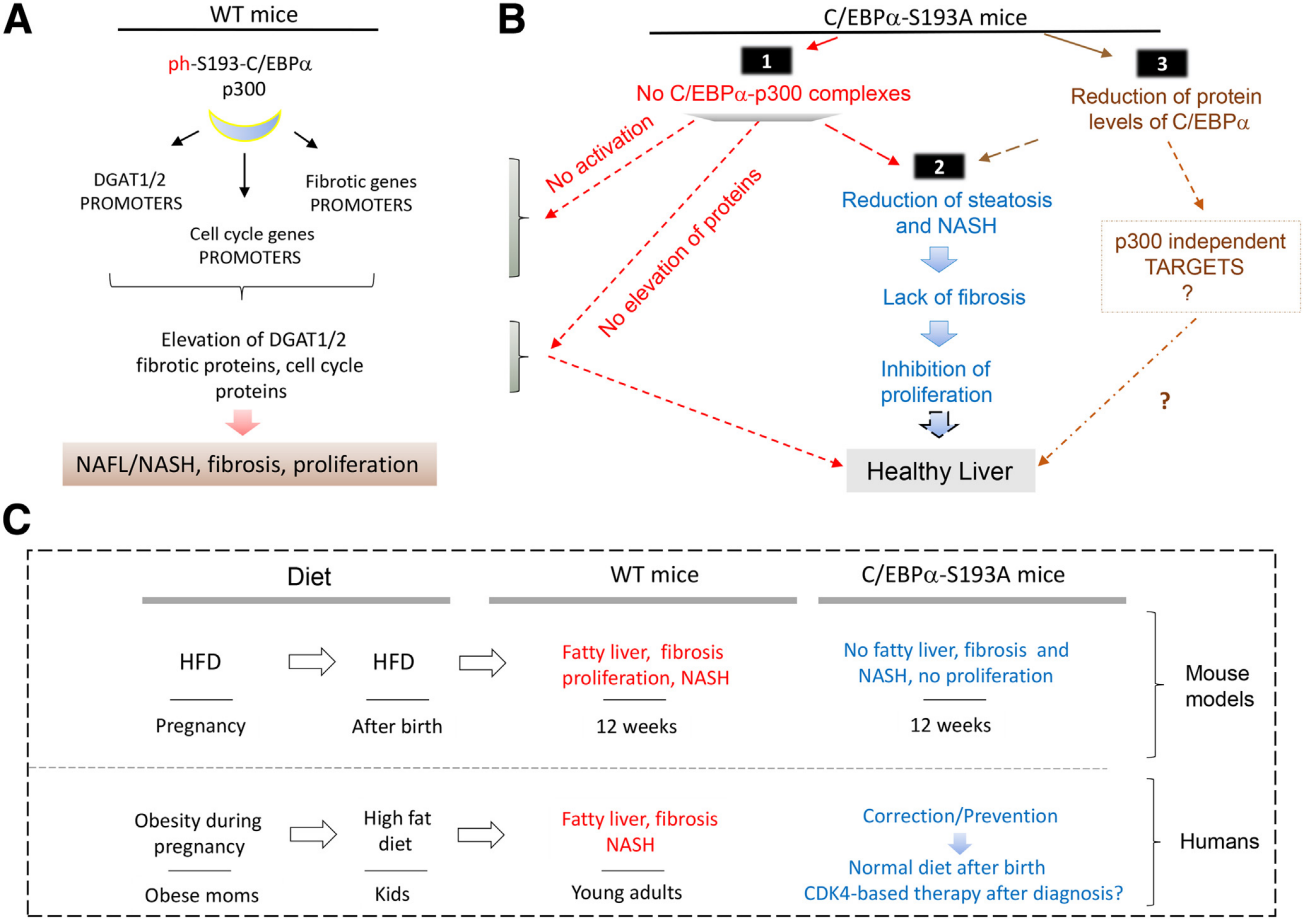
**Obese pregnancy–associated pathways of liver disorders in offspring that are inhibited in livers of C/EBPα-S193A mice.** (*A*) WT mice: pathways of obese pregnancy–dependent liver disorders in HH arm of WT mice. Downstream targets of C/EBPα-p300 complexes are shown. (*B*) C/EBPα-S193A mice: molecular pathways and biological processes that are inhibited in livers of HH arm of C/EBPα-S193A mice. *Numbers 1, 2, and 3* show 3 pathways that were inhibited in obese-pregnancy HH arm of C/EBPα-S193A mice and that contribute to inhibition of liver disorders in offspring. (*C*) Translational aspects of studies of offspring of pregnancy-obese mice to liver disorders observed in obese children.

Our studies on C/EBPα-S193A mice, which have a single amino acid substitution, revealed a robust inhibition of fatty liver, NASH, fibrosis, and liver proliferation that developed in the offspring of WT mice in the HH arm. This strong inhibition was surprising and raised the question of how a single amino acid substitution in the C/EBPα molecule might cause such a strong inhibition of several liver diseases. We suggest that several mechanisms might be involved and propose 3 scenarios (Figure 7). First, the main cause of the inhibition of liver disorders in C/EBPα-S193A offspring in the HH arm is the mutation of Ser193 to Ala and the subsequent lack of C/EBPα-p300 complexes. We learned that the C/EBPα-p300 complexes activate expression of multiple genes that are involved in the development of liver disorders: fatty liver development, NASH, fibrosis, and increased liver proliferation. Therefore, the lack of the C/EBPα-p300 complexes in C/EBPα-S193A mice in the HH diet arm may be associated with the lack of activation of genes coding for fatty liver, fibrotic, and cell cycle proteins, resulting in the inhibition of these liver diseases independently of each other (Figure 7*B*). A second possible mechanism is likely associated with cross-interactions of biological processes in the liver. It has been shown by many reports that steatosis can lead to a liver injury, which in turn increases liver proliferation as has been shown in CCl4-mediated liver injury.^26^ Therefore, it is likely that the lack of steatosis in the C/EBPα-S193A offspring of the HH arm contributes to the lack of fibrosis, resulting in the reduction of liver proliferation (Figure 7*B*). A third mechanism is related to the fact that total levels of C/EBPα are reduced in C/EBPα-S193A mice (Figure 4*A* and *C*). In addition to complexes with p300, C/EBPα regulates expression of genes via formation of complexes with other chromatin remodeling proteins such as histone deacetylase 1 (*HDAC1)*.^15^^,^^18^ Moreover, we have recently found that human de-ph-Ser190-C/EBPα (human S190 is an analog of mouse S193 and de-phosphorylation on this residue mimics the S193A mutation) forms complexes with HDAC1, and that these C/EBPα-HDAC1complexes down-regulate hepatocyte specific genes in liver cancer.^28^ Therefore, the reduction of total levels of C/EBPα might result in additional p300 independent epigenetic changes. In summary, we conclude that the main pathway of inhibition of liver disorders in offspring of HH arm of C/EBPα-S193A mice is the lack of C/EBPα-p300 complexes; however, additional indirect pathways are also involved (Figure 7*B*).

## Discussion

The initial stages of development of obesity-associated liver diseases in children and adolescents are mainly silenced because of minor or no symptoms. However, several recent studies clearly indicated that maternal obesity and HFD in children are the key reasons for liver disorders in adolescents. These disorders include NAFLD, fibrosis, and liver cancer. Our long-time studies of pediatric liver cancer, HBL, provided us with several examples where HBL was associated with severe fatty liver and with fibrosis. We also observed that dysregulation of epigenetic control of gene expression is correlated with a fatty liver phenotype in adult mice.^16^^,^^18^^,^^19^ In this article, we examined the contribution of C/EBPα-dependent epigenetic activities in the development of pregnancy-OWOB–mediated liver disorders. The ability of mouse C/EBPα to activate or repress target genes depends on the phosphorylation at Ser193 (Ser190 in human protein). Upon phosphorylation at Ser193, C/EBPα interacts with histone acetyltransferase p300, activating genes involved in glucose metabolism, fatty liver, fibrosis, and liver cancer.^16^^,^^19^^,^^24^^,^^25^ Studies in this article clearly demonstrated the critical role of ph-S193-C/EBPα in maternal obesity-associated fatty liver, as well as fibrosis and increased proliferation. Our data show that 3 main biological processes (liver diseases) are under control of epigenetic activities of C/EBPα in the settings of prenatal HFD and subsequent postnatal HFD. These processes include steatosis, fibrosis, and liver proliferation. Although our molecular studies revealed that genes involved in all these processes are directly controlled by C/EBPα, we believe that there might also be connections between these disorders and pathways that are outside of direct C/EBPα control. The existence of other pathways is highly likely because HFD-resistant C/EBPα-S193A mice have a single amino acid substitution, but the inhibition of liver disorders is dramatic. One of the possible pathways is a crosstalk between biological processes. Although fibrotic genes and cell cycle genes can be directly regulated by C/EBPα, it is likely that the lack of steatosis in the HH arm of C/EBPα-S193 mice contributes to the reduced NASH and fibrosis. It is also likely that other transcription factors and chromatin remodelers might be involved in the inhibition of liver disorders in HH arm of C/EBPα-S193A mice. In this regard, it is interesting to mention that another member of the C/EBPα family, C/EBPβ, is highly expressed in the liver and binds to C/EBP consensuses identical to those of C/EBPα.^29^ It has been shown that C/EBPα is involved in the development of CCl4-mediated fibrosis in adult mice.^26^ Given identified epigenetic alterations on the C/EBP sites of genes of fatty liver, fibrosis and proliferation (Figure 4, Figure 5, Figure 6), it will be important to examine whether C/EBPα might also contribute to HFD during pregnancy associated-liver disorders in offspring.

Several NAFLD studies using an HFD found that liver proliferation is the first event that takes place after the start of the diet before the appearance of liver steatosis, suggesting liver proliferation could be involved in progressing NAFLD, including fibrosis.^16^^,^^21^^,^^30^ In addition, elevation of cell cycle proteins and oncogenes has been reported in biopsies from patients and in animal models with NAFLD. These cell cycle proteins and oncogenes include cyclin dependent kinase CDK4,^16^^,^^19^ Gankyrin,^31^ Cyclin D3,^20^^,^^25^ and E2F1.^32^, ^33^, ^34^ This article demonstrates that feeding pregnant females and their subsequent offspring with HFD causes elevation of CDK4 in the livers of the offspring (Figure 4), which has been reported to be elevated in NASH patients.^19^ On the basis of these observations and because liver proliferation is high during prenatal and early postnatal stages of liver development, we suggest that the initiation of liver proliferation in the HH arm of WT mice might be a causal event in the development of fibrosis. Consistent with this hypothesis, we observed a strong opposite correlation between levels of steatosis and proliferation in many areas of fatty HBL specimens, as well as in the livers of WT mice in the HH arm (data not shown).

How can our studies in mouse models be translated to humans? We have presented our hypotheses in Figure 7*C*. In mouse models, it was found that the most dangerous combination of diet is the HFD during pregnancy and subsequent HFD after birth. It appears that a low-fat diet after birth may reduce or eliminate the consequences of prenatal obesity in young children. Taken together, these studies determined that epigenetic mechanisms are not only observed in liver disorders from the offspring of an OWOB pregnancy but may cause the progression of these liver disorders. This knowledge can be used for the prevention of liver disorders in children by focusing on an appropriate diet, as well as by the development of potential therapeutics for obese children with NAFLD symptoms. Regarding possible regimens, the triggering event of obese pregnancy–associated liver disorders is phosphorylation of C/EBPα at Ser193 by CDK4. Because CDK4 is a strong promoter of liver proliferation, we propose that the inhibition of liver proliferation in children with NASH/NAFLD could be considered as a potential therapy. The therapeutic approach with targeting liver proliferation is also supported by several reports showing that cell cycle proteins CDK4, Cyclin D3, E2F1, and liver cancer promoting protein Gankyrin play a critical role in development of NAFLD.^19^^,^^20^^,^^32^, ^33^, ^34^ Further studies are required to test this hypothesis.

## Methods

### Animal Work

Experiments with animals were approved by the IACUC at Cincinnati Children’s Hospital (protocols IACUC2014-0042, IACUC2017-0041, and IACUC2020-0049). C/EBPα-S193A and C/EBPα-S193D knock-in mice have been previously characterized in our lab.^18^, ^19^, ^20^ WT siblings from the C/EBPα-S193D strain (previously characterized in our lab) were used as WT controls. Figure 2*A* shows the strategy for the treatments of WT and C/EBPα-S193A knock-in mice. The following treatments were aligned for 4 arms. The first arm included treatments of pregnant females and then subsequent pups at weaning with normal chow (NN arm). This arm is the control reflecting healthy conditions. The second arm included treatments of pregnant females with normal chow and subsequent treatments of pups with HFD (60% HFD, Bio-Serv S3282) at weaning (NH arm). This arm was designed to monitor the after-birth effects of HFD on liver disorders in offspring. The third arm included HFD treatments of pregnant females, followed by treatments of weaned pups with normal diet (HN arm). The fourth arm included treatments of pregnant females with HFD, followed by treatments of weaned pups with HFD (HH arm). This arm recapitulates the human condition where children of obese mothers had high-fat food after birth.

The animals of all 4 arms were killed 16 weeks after birth, and livers were examined with the focus on liver disorders: fatty liver, fibrosis, proliferation, and NASH as described below. Three to 12 mice were analyzed in each arm of WT and C/EBPα-S193A mice. Two to 3 C/EBPα-S193D mice were analyzed for development of steatosis. We also examined the effect of prenatal HFD on body weight in males and females. This examination of different genders did not show differences in the outcomes. The identical inhibition of steatosis and fibrosis was observed in males and females of high-fat/high-fat arm of S193A mice. Quantitation of hepatic steatosis was performed by counting number of fat droplets per field at ×10 magnification. Ten fields of livers of 3 mice of each genotype were used for calculations.

### Histology, ki67, and Sirius Red Staining

The livers were fixed overnight in 4% paraformaldehyde, processed to 70% EtOH in saline, embedded in paraffin, and sectioned at 7 μm. Slides were stained with hematoxylin-eosin (ab245880, Abcam) for histologic examination. For ki67 (ab16667, Abcam) staining, slides were deparaffinized and rehydrated through an ethanol gradient to 1× phosphate-buffered saline. Antigen retrieval was performed at high pressure and high temperature in sodium citrate pH 8 buffer. The samples were blocked for 1 hour at room temperature. The samples were then incubated overnight at 4°C with primary antibody diluted in blocking buffer. The next day, the samples were washed and incubated with secondary antibody diluted in blocking buffer for 4 hours at room temperature, counterstained with Mayer’s hematoxylin, dehydrated through an ethanol series, and mounted with Cytoseal 60 (Thermo Scientific, 8310-4). The percentage of ki67-positive hepatocytes was calculated by manually counting five ×20 image fields from an animal (n = 3 for each arm).^18^^,^^20^ Sirius Red staining was performed using the Picosirius Red Stain Kit from Polysciences (24901) following manufacturer’s instructions. Images were taken using a Nikon Eclipse 90i Microscope and a Nikon Eclipse Ti Microscope.

### Real-Time Quantitative Reverse Transcriptase Polymerase Chain Reaction

Total RNA was isolated using the TRIzol method, and cDNA was synthesized using Applied Biosystems High-Capacity RNA-to-cDNA kit and diluted as previously described.^19^^,^^20^ TaqMan probes for *DGAT1* (Hs01017541_m1), *DGAT2* (Hs01045913_m1), *FASN* (Hs01005622_m1), *SCD* (Hs01682761_m1), *COL1A1* (Hs00164004_m1), *COL1A2* (Hs01028956_m1), and *18s* (Hs03003631_g1) were purchased from Applied Biosystems (Foster City, CA) and used with TaqMan Gene Expression mastermix (Applied Biosystems, 4369016). Relative levels of mRNA transcripts were calculated using the delta-delta Ct method, normalizing to *18s* levels with fold-change relative to the background liver sample for each patient.

#### Protein Isolation and Western Blot

Whole cell extracts and nuclear extracts were isolated from livers as described in previous articles.^16^, ^17^, ^18^, ^19^, ^20^^,^^24^^,^^25^ Fifty μg of proteins were loaded on gradient (4%–20%) polyacrylamide gels (Bio-Rad), transferred onto nitrocellulose membranes, and probed with antibodies against proteins of interest. To verify protein loading, membranes were re-probed with an antibody to β-actin. Antibodies to C/EBPα (14AA), DGAT1 (H-255), DGAT2 (H-70), cyclin D1 (H-295), cdk4 (C-22), and β-actin (C4) were from Santa Cruz Biotechnology (Dallas, TX). Antibodies to Gankyrin were from (Cell Signaling Technologies, 12985 S). The intensities of the protein bands were calculated using Java-based image processing software ImageJ. The results are presented as ratios of signals of proteins to signals of the loading control β-actin.

### Co-immunoprecipitation

Whole cell extracts and nuclear extracts were used for co-immunoprecipitation as described in our previous articles.^16^, ^17^, ^18^, ^19^, ^20^^,^^24^^,^^25^ TrueBlot reagents were used as previously described with a few modifications. The modified steps are as follows. The immunoprecipitations were resuspended in 30 μL of loading buffer containing 2% sodium dodecyl sulfate and 5 mmol/L β-mercaptoethanol and boiled for 40 minutes. Ten μL of the samples was run on sodium dodecyl sulfate gel (4%–20% gradient gels, Bio-Rad). True-Blot mouse and rabbit beads and secondary antibodies were used.

### Pull-down Assay

The main strategy for this assay is shown in Figures 4*D* and 5*D*. Complementary biotinylated ssDNA oligomers (40–60 nucleotides) containing consensuses for C/EBPα were ordered from Integrated DNA Technologies and annealed to make dsDNA oligomers and linked to Dynabeads M-280 Streptavidin magnetic beads (Invitrogen, 11205D). The sequences of upper strains are as follows: *Mmp13* (5′-biotin- GG CAC TGT AGT GCA TTT CTC AAA TCC CTA TTC TCT TAA AAG-3′), *Tgfb1* (5′-biotin- AA GAC CAT CGA CAT GGA GCT GGT GAA ACG GAA GCG CAT CGA AGC CAT-3′), *Tnf* (5′-biotin- GG ATG TCC CAT TTA GAA ATC AAA AGG AAA TAG ACA CAG GCA TGG TCT TTC TAC AAA GA-3′), *Hmmr* (5′-biotin-GA CAC TCG TGA GCC CTG GTG AAA TCG ACC GAA GCC TAG AG-3′), *Cdk1* (5′-biotin-AG TAC GGT AGT GTG CTG AAA TTC AGC CCT GGG GAT CA-3′), and *Ccdn1* (5′-biotin- CT GAA TTT TAC ACG TGT TGA AAT TGA AAG ACA GG-3′). The streptavidin-dsDNA-oligomers were incubated with nuclear proteins from livers of HH arms of WT and C/EBPα-S193A mice in buffer containing 10 mmol/L Tris-HCL pH 7.5, 150 mmol/L NaCl, 5 mmol/L β-mercaptoethanol, and 10% glycerol for 4 hours. The beads were washed with the buffer 3 times, and proteins were eluted using sodium dodecyl sulfate loading buffer. The eluted proteins were further analyzed by 4%–20% sodium dodecyl sulfate–polyacrylamide gel electrophoresis Western blotting with antibodies to C/EBPα and p300.

### Chromatin Immunoprecipitation

ChIP was performed as described in our previous articles.^19^^,^^20^ Chromatin solutions were prepared from livers of HH arms of WT mice and C/EBPα-S193A mice. Primer sequences for *Dgat1* and *Dgat2* promoters can be found in our articles.^24^^,^^25^ Primer sequences for ChIP with others genes are as follows: *Mmp13* first intron (F: 5′-ATGGTGATGATGATGATGATGAC-3′, R: 5′-AAGCCAAAGAAAGATTGCATTTC-3′); *Tgfb1* first intron (F: 5′-AAGACCATCGACATGGAGC-3′, R:5′-ATTAGCACGCGGGTGAC-3′); *Tnf* promoter (F: 5′-CCTCAATATCATGTCTCCCCC-3′, R: 5′-GCCAATCAGGAGGGTGTG-3′); *Ccdn1* promoter (F: 5′-AAAGTTCAGATACCCCTCTGGCCC-3′, R: 5′-GGGGGTGCAAGGACACCCTG-3′); *Cdk1* promoter (F: 5′-CGAGGCCACAGGTTGGCC-3′, R: 5′-CAAGCAGGCCTGTCAAGCCG-3′); and *Hmmr1* promoter (F: 5′-GACTAGGCTCTGGGCTTGCCC-3′, R:5′-ACAGGCCGGTGGACGCGTC-3′). Polymerase chain reaction conditions were as follows: 99°C for 5 minutes, followed by 35 cycles of 94°C for 30 seconds, 60°C for 1 minute, 72°C for 30 seconds, and a final extension step at 72°C for 10 minutes. The polymerase chain reaction products were analyzed by 6%–8% polyacrylamide gel electrophoresis.

### Liver Triglyceride and Serum Triglyceride Assays

Liver TG assays were performed as previously described.^19^ Briefly, 100 mg of frozen liver tissue was homogenized in 1 mL of homogenization buffer (50 mmol/L Tris-HCL pH 7.5, 150 mmol/L NaCl, 1 mmol/L EDTA, and 1 mmol/L PMSF) and incubated on ice for 30 minutes. The supernatant was collected and diluted 1:10 in homogenization buffer. Ten μL of diluted supernatant for each sample and a set of TG standards (Pointe Scientific, T7531-STD) were loaded in triplicate on a 96-well plate. To each well, 200 μL of Triglyceride Reagent (Pointe Scientific, T7531-120mL) was added, and the plate was incubated for 5 minutes at 37°C. Absorbance was measured on a plate reader at 500 nm, and TG concentration for each sample was calculated on the basis of the standard curve. Protein concentration for each sample was calculated using Pierce 660nm Protein Assay Reagent (Thermo Scientific, 22660) according to manufacturer’s instructions, and the concentration of TG was calculated per mg of protein. Serum TGs were measured with the Pointe Triglyceride Set (T7531-STD). Serum samples were diluted 1:5 in 0.9% saline. Ten μL of diluted serum samples and a set of standards were loaded in triplicate on a 96-well plate. To each well, 200 μL Triglyceride Reagent was added, and the plate was incubated for 5 minutes. Absorbance was measured at 500 nm, and serum TG concentration for each sample was calculated on the basis of the standard curve.

### Statistical Analysis

All continuous values are presented as mean ± standard deviation using Microsoft Excel (Redmond, WA) and GraphPad (La Jolla, CA) Prism 9.0. Student *t* tests, one-way analysis of variance, and two-way analysis of variance were performed as appropriate, and *P* < .05 was considered significant.
